# The preventing recurrent vascular events and neurological worsening through intensive organized case-management (PREVENTION) trial protocol [clinicaltrials.gov identifier: NCT00931788]

**DOI:** 10.1186/1748-5908-5-27

**Published:** 2010-04-12

**Authors:** Finlay A McAlister, Sumit R Majumdar, Rajdeep S Padwal, Miriam Fradette, Ann Thompson, Ross Tsuyuki, Steven A Grover, Naeem Dean, Ashfaq Shuaib

**Affiliations:** 1 Division of General Internal Medicine, University of Alberta Hospital, 8440 112 Street, Edmonton, Canada; 2 Mazankowski Alberta Heart Institute, University of Alberta, 8440 112 Street, Edmonton, Canada; 3 The Epidemiology Coordinating and Research (EPICORE) Centre, University of Alberta, 220 College Plaza, Edmonton, Canada; 4 Provincial Pharmacy Services, Alberta Health Services, University of Alberta Hospital, 8440 112 Street, Edmonton, Canada; 5 McGill Cardiovascular Health Improvement Program (CHIP), Division of General Internal Medicine, McGill University, Montreal, Canada; 6 Division of Internal Medicine, Royal Alexandra Hospital, 10240 Kingsway Avenue, Edmonton, Canada; 7 Division of Neurology, University of Alberta Hospital, 8440 112 Street, Edmonton, Canada

## Abstract

**Background:**

Survivors of transient ischemic attack (TIA) or stroke are at high risk for recurrent vascular events and aggressive treatment of vascular risk factors can reduce this risk. However, vascular risk factors, especially hypertension and high cholesterol, are not managed optimally even in those patients seen in specialized clinics. This gap between the evidence for secondary prevention of stroke and the clinical reality leads to suboptimal patient outcomes. In this study, we will be testing a pharmacist case manager for delivery of stroke prevention services. We hypothesize this new structure will improve processes of care which in turn should lead to improved outcomes.

**Methods:**

We will conduct a prospective, randomized, controlled open-label with blinded ascertainment of outcomes (PROBE) trial. Treatment allocation will be concealed from the study personnel, and all outcomes will be collected in an independent and blinded manner by observers who have not been involved in the patient's clinical care or trial participation and who are masked to baseline measurements. Patients will be randomized to control or a pharmacist case manager treating vascular risk factors to guideline-recommended target levels. Eligible patients will include all adult patients seen at stroke prevention clinics in Edmonton, Alberta after an ischemic stroke or TIA who have uncontrolled hypertension (defined as systolic blood pressure (BP) > 140 mm Hg) or dyslipidemia (fasting LDL-cholesterol > 2.00 mmol/L) and who are not cognitively impaired or institutionalized. The primary outcome will be the proportion of subjects who attain 'optimal BP and lipid control'(defined as systolic BP < 140 mm Hg and fasting LDL cholesterol < 2.0 mmol/L) at six months compared to baseline; 12-month data will also be collected for analyses of sustainability of any effects. A variety of secondary outcomes related to vascular risk and health-related quality of life will also be collected.

**Conclusions:**

Nearly one-quarter of those who survive a TIA or minor stroke suffer another vascular event within a year. If our intervention improves the provision of secondary prevention therapies in these patients, the clinical (and financial) implications will be enormous.

## Background

Epidemiological studies have shown that a number of common conditions increase the risk of stroke and other vascular diseases, and there is compelling evidence from large randomized trials that treatment of such conditions, especially hypertension and high cholesterol, can lead to a significant reduction in the incidence and recurrence of cardiovascular events [1-6]. We know that vascular risk factors, in particular hypertension and high cholesterol, are not managed optimally in patients after stroke or transient ischemic attack (TIA), even in those patients seen in specialized stroke prevention clinics (SPCs) [7,8]. The recent Canadian best practice recommendations for stroke care [9] highlighted this care gap and emphasized that 'new strategies to support clinical practice...are urgently needed'. Implementation of such strategies is important because the risk of recurrent vascular events is high in patients who have suffered a stroke or TIA (*e.g*., in a recent study of 2,285 TIA survivors in Alberta, the rate of stroke, myocardial infarction (MI), or death at one year was 22%) [10]. Stroke prevention strategies need to extend beyond the use of anti-platelet agents, anticoagulation, and carotid endarterectomy, and start addressing the need for more aggressive management of the key stroke risk factors of hypertension and hyperlipidemia, as well as consideration of other modifiable factors such as medication adherence, smoking cessation, diabetes, exercise, and weight loss.

### Current stroke prevention practices

A recent analysis of data on over 2,000 patients evaluated in the SPC at the University of Alberta Hospital (Edmonton, Canada) confirmed the suboptimal management of vascular risk factors in patients with recent stroke or TIA in our health region [7,8]. Treatable risk factors (hypertension and dyslipidemia) for stroke were seen in over 80% of patients, and the vast majority of these patients were not controlled to recommended targets at any point during the first year after being seen in our SPC [8]. Furthermore, there did not seem to be any substantial improvement in risk factor management in our health region over the four years encapsulated within the pilot data collection [7,8], nor in the intervening three years since their publication. An analysis of the electronic database maintained by Alberta Health Services for SPC patients up to July 2008 revealed that 77% of patients at baseline (and 68% at follow-up) did not meet currently recommended low-density lipoprotein (LDL) cholesterol targets for stroke/TIA patients of ≤2.0 mmol/L (Dr. Thomas Jeerakathil, Chair of the Evaluation and Quality Improvement Pillar of the Alberta Provincial Stroke Strategy, personal communication, August 18, 2008), suggesting little change will occur outside of a targeted intervention.

It should be acknowledged that our current stroke prevention system (comparable to other SPCs in Canada) is largely consultative in that the stroke specialists provide written recommendations to each patient's family physician but infrequently initiate or up-titrate blood pressure (BP) or lipid-lowering medications, and SPC nurses are not involved in medication adherence strategies or telephone follow-up of this population. As such, patients are 'falling through the cracks' in our current system of care. Our experience is virtually identical to that reported from other SPCs [11]. Indeed, the under-treatment of vascular risk factors is not restricted to stroke specialists and their patients; it is also seen for other atherosclerotic conditions [12-14]. The recently reported EXPRESS study demonstrated (in a controlled before-after design) that prompt initiation of various secondary prevention manoeuvres in a British SPC (compared to their prior practice of merely recommending therapy to primary care physicians) resulted in substantial improvements in risk factor management within the first month after TIA, and an 80% reduction in recurrent stroke within three months [15].

### The crucial role of hypertension and lipid management for secondary prevention

Hypertension is the most important modifiable risk factor for vascular disease; approximately 22% of adult Canadians have hypertension [16], and it is the most common attributable cause for mortality in developed nations [17]. Three-quarters (74%) of our SPC attendees had a diagnosis of hypertension in our pilot study [7,8], almost identical to the 71% prevalence of hypertension in a large community-based cohort study of elderly US stroke survivors [18]. There is a strong log-linear relation between BP levels and vascular outcomes in both genders, across all age strata, and in all ethnic groups [3]; of particular relevance to this proposal, lowering of systolic BP by 5 mm Hg has been shown consistently to confer a 20% to 25% reduction in stroke rates (in both primary and secondary prevention) which accrues relatively quickly (within two years) [3,19,20]. Importantly, the benefits of antihypertensive therapy in stroke/TIA survivors enrolled in the PROGRESS trial were seen across all quartiles of baseline BPs (including the lowest quartile in which median baseline systolic pressures were 114 mm Hg), with no evidence of a J-curve relationship [21]. In addition, a recent analysis of 26 trials of angiotensin converting enzyme (ACE) inhibitors demonstrated blood-pressure independent reductions in cardiovascular events, even in patients with baseline systolic BPs of less than 140 mm Hg [22]. The current Canadian best practice recommendations for stroke care [9] advocate the use of ACE inhibitors as first-line therapy and recommend target systolic BPs of < 140 mm Hg in patients after the acute phase of stroke or TIA, or < 130 mm Hg in those with concomitant diabetes or chronic kidney disease. On the basis of the meta-analysis [22] of 26 ACE inhibitor trials and the secondary analyses from PROGRESS [21], PRoFESS [23], and TRANSCEND [24] demonstrating similar benefits accrued from renin-angiotensin system inhibition, regardless of baseline BP, it seems reasonable to initiate renin-angiotensin system inhibition if the systolic BP exceeds 130 mm Hg, a treatment strategy also used in the EXPRESS Study [15] and consistent with both arms of the ongoing SPS3 trial (Secondary Prevention of Small Subcortical Strokes Trial; clinicaltrials.gov identifier: NCT00059306).

Similar data is becoming available confirming the efficacy of statins in the primary and secondary prevention of ischemic stroke [4,5,25]. Importantly, analogous to the previous discussion regarding ACE-inhibition for hypertension treatment, statins appear to be beneficial regardless of baseline LDL cholesterol, and the magnitude of benefit is directly related to the degree of LDL cholesterol reduction achieved [4,5,26]. Furthermore, in a recent meta-analysis of all high-dose versus low-dose statin trials (seven trials, 29,395 patients), we demonstrated an additional 18% relative reduction in stroke (95% CI 5% to 29%) with high-dose statins [27].The current Canadian best practice recommendations for stroke care [9] advocate the use of statins in all patients after a stroke or TIA, with a target goal of < 2.0 mmol/L LDL cholesterol.

### Potential methods for improving secondary prevention after stroke/TIA

It has consistently been shown that multiple barriers (patient-, physician-, and healthcare system-related) are responsible for the lack of implementation of proven efficacious therapies and traditional means of educating practitioners (*e.g*., journal articles, continuing medical education conferences, grand rounds lectures) are usually ineffective in altering practice. We have previously outlined the rationale for our research program and highlighted various potential knowledge translation strategies that may enhance the provision of evidence-based care but which require evaluation [28]. Over the past five years, our group has tested a number of these strategies in randomized trials, including patient decision aids for atrial fibrillation [29], multidisciplinary teams for patients with diabetes [30] and osteoporosis [31], pharmacy screening programs and reminder programs for patients with angina and their primary care physicians [32], and local opinion leader-based interventions for patients with heart failure [33], hypertension [34], and coronary artery disease [35]. In consultation with the stroke neurologists and opinion leaders in stroke management in Alberta (through the health promotion and prevention pillar of the Alberta provincial stroke strategy), and taking into account the results of our prior knowledge translation studies, we developed a pharmacist case manager intervention for secondary prevention after stroke/TIA that we will evaluate in this trial

### Why test a pharmacist case manager for secondary prevention after stroke?

Disease management programs that utilize a systematic and multidisciplinary approach (usually nurse- or pharmacist-based) for secondary prevention in patients with ischemic heart disease have demonstrated substantial improvements in atherosclerotic risk factors and significant reductions in the incidence of recurrent disease in many, but importantly, not all studies [36,37]. While 11 randomized trials have demonstrated that nurse or pharmacist case managers who made medication adjustments achieved better glycemic control in patients with type 2 diabetes than usual care by physicians or passive case managers, who could only highlight the need for medication titration to each patient's primary care physician [38], whether these benefits extend to other conditions remains uncertain and is the premise of this trial. Although several quasi-experimental studies or randomized trials with surrogate process outcomes (such as having LDL measured) suggest that pharmacist case management would be beneficial [39-46], the evidence on clinical outcomes (such as changes in BP or LDL cholesterol levels or rates of recurrent MI, stroke, or death) is sparse and inconsistent between studies. Indeed, a recently published systematic review of this literature found that only 57% of studies suggested benefit with this intervention, and the quality of the studies was such that the authors concluded 'more high-quality studies are needed' [46]. Perusal of the third issue of the 2009 Cochrane Library reveals two current Cochrane reviews on this topic. One concluded that 'health professional (nurse or pharmacist) led care appears to be a promising way of delivering care but requires further evaluation'[47], while the other concluded that 'the question of whether pharmacists can manage drug therapy as well as physicians remains unanswered due to a shortage of studies...more rigorous research is needed'[48]. Perusal of the 'Closing the Quality Gap' series on the Agency for Healthcare Research and Quality (AHRQ) website confirmed that the intervention we have proposed to test is promising but unproven [49]. Thus, although the use of a pharmacist case manager to target secondary prevention in patients with TIA or stroke holds great promise, as of yet this is a promise unfulfilled, and a hypothesis which needs to be tested.

## Methods

### Study design

This study will compare the intervention (pharmacist case managers treating cardiovascular risk factors to target levels employing standardized protocols) to control group (which, as detailed below, actually represents an enhancement over usual care) utilizing a prospective, randomized, controlled open-label with blinded ascertainment of outcomes (PROBE) design. The individual patient will be the unit of allocation, the unit of analysis, and the unit of causal inference.

### Study setting

All three SPCs in Edmonton, Canada (population 1.1 million people) are participating in this trial.

### Study participants

Patients older than 18 years of age, with a stroke or TIA, who are evaluated at a SPC are eligible for the study if they are confirmed by a stroke specialist to have had an ischemic stroke or TIA within the past year and have systemic hypertension (average systolic BP over two visits exceeding 140 mmHg), fasting LDL cholesterol exceeding 2.0 mmol/L, or total:high-density lipoprotein (HDL) cholesterol ratio exceeding 4.0.

Patients will be excluded if any one of the following criteria apply:

1. Neurological event considered to be due to hemorrhage (*e.g*., intracranial hemorrhage, subarachnoid hemorrhage), cardiac embolus related to structural heart disease (valve abnormality, atrial or ventricular septal defect, endocarditis), or trauma (as defined by stroke specialists).

2. Participation in a concurrent trial related to stroke or vascular disease.

3. Any condition that would preclude treatment benefit or 12-month follow-up, including foreshortened life-expectancy (*e.g*., active malignancy), hypertensive urgency (clinic systolic BP ≥200 mm Hg), or severe comorbidities.

4. Institutionalized in a long-term care facility.

5. Impaired cognition (scored ≥5 on the Short Portable Mental Status questionnaire) [50].

6. Refractory hypertension or hyperlipidemia (levels uncontrolled despite already being on three antihypertensive drugs at maximal dose if hypertension is their inclusion criterion, or on maximal dose statin if hyperlipidemia is their inclusion criterion).

### Experimental arms

#### Control

Calling our control arm 'usual care' would be a misnomer, because it refers to usual care provided via specialized SPC and monthly visits with a study nurse. All three SPCs that will enroll patients have standardized approaches, protocols, and guidelines with respect to definitions and diagnoses of stroke and TIA; routine investigations to determine potential benefit of surgical approaches; routine application of anti-platelet medications; and routine suggestions to primary care physicians with respect to vascular risk factors such as hypertension, dyslipidemia, smoking, and other lifestyle issues. However, similar to other jurisdictions, our SPC current practice, confirmed in our pilot data [7,8] suggests that these clinics do not undertake active ongoing management of vascular risk factors but rather instead provide suggestions and directions to each patient's primary care physician. Patients randomized to the control arm will receive the same educational materials about stroke risk factors and medication adherence as the intervention patients, will be seen monthly by a study nurse, will have the same number of BP measurements, and will have a fax sent to their family physician after each study visit reporting their BP and current medications. Of note, our control arm actually represents the intervention used in our recently published SCRIP-HTN trial in individuals with diabetes which resulted in a 5 mm Hg reduction in systolic BPs compared to usual care (p = 0.03) [34]. Thus, some might consider our study to be an active-comparator trial.

#### Intervention

Over and above usual care, our intervention will include intensive pharmacist case-management, consisting of monthly follow-up visits with the study pharmacist for six months that will be independent of any planned follow-up with the SPC or family physicians. At each visit, the study pharmacist will monitor the patient's BP and lipid levels and will initiate and/or titrate antihypertensive and/or hypolipidemic therapy as appropriate. The study pharmacist will follow treatment algorithms consistent with current Canadian national guidelines [51,52]. The pharmacist will emphasize medication and lifestyle adherence with patients and their caregivers, using the cardiovascular risk profile as an educational aid as per prior studies by our group [53,54]. The pharmacist will also send a fax to the primary care physician after each study visit outlining the status of that patient's atherosclerosis risk factors and any therapy adjustments made at that visit.

### Study procedures (Figure 1)

**Figure 1:**
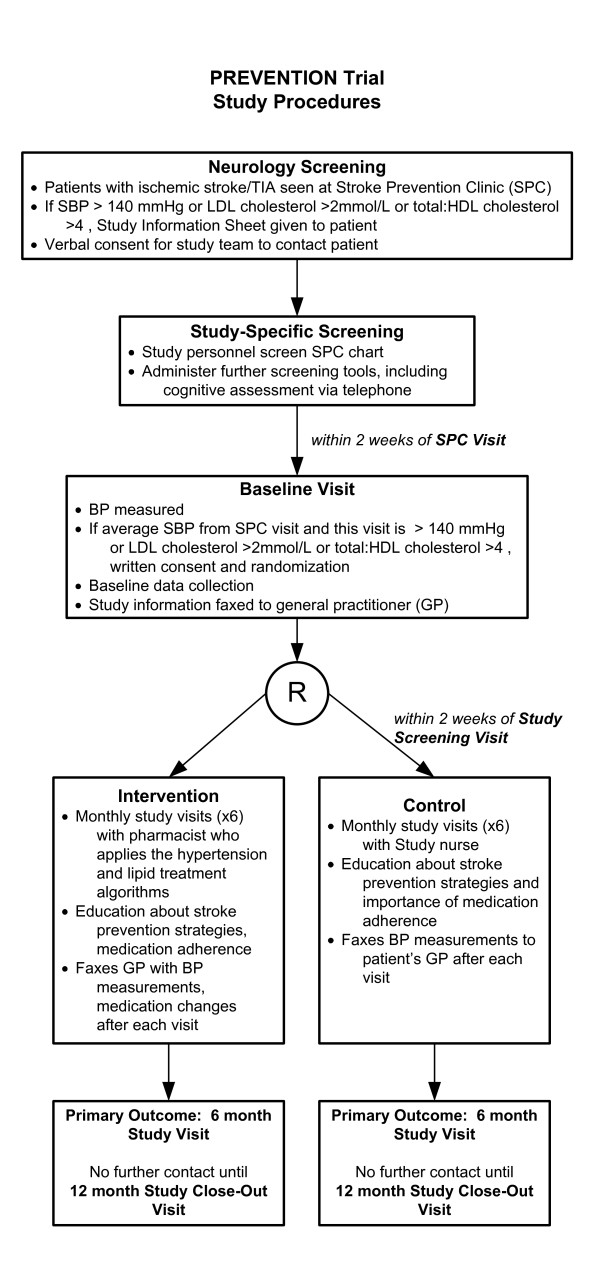
**Prevention Trial Study Procedures**. TIA = transient ischemic attack, SPC = stroke prevention clinic, SBP = systolic blood pressure, GP = general practitioner.

#### Screening

After initial assessment in participating SPCs (which will include standard SPC workup, including collection of data elements for the ABCD^2 ^score [55], carotid dopplers, CT scan, fasting lipid profile, fasting glucose, electrolytes, creatinine, complete blood count (CBC), liver function tests, glycosylated hemoglobin, and electrocardiogram), all potentially eligible patients who consent to further screening for the trial will be assessed in person at a study screening visit within two weeks by a research assistant. At this visit, they will have their BP measured using the BpTRU^® ^device (VSM MedTech, Vancouver, BC, Canada). If their average systolic pressure (averaged between the SPC measurement and the average measurement at the study screening visit) exceeds 140 mm Hg or their fasting lipid profile reveals LDL cholesterol exceeding 2.0 mmol/L or total:HDL cholesterol ratio > 4, and they provide written consent for the study, baseline case report forms will be completed.

#### Randomization

Patients will be randomized 1:1 to control or intervention. Randomization will be done centrally by computer-generated random numbers, and a secure internet-based allocation method that ensures allocation concealment from all research personnel; randomization will be stratified by participating SPC. As this study is unblinded, variable sized blocked randomization will also be used to preserve allocation concealment. Because our intervention is distinct from usual care and current standards of care in participating clinics are well demarcated, the risk of contamination (by altering the practices of physicians in participating SPCs) is low. Furthermore, our pilot data suggests that the ratio of patients enrolled to primary care physicians affected in this study is essentially 1:1--that is, it is unlikely that any one of the almost 950 primary care physicians in Edmonton will have more than one patient enrolled in this study. Thus, the patient will be the unit of randomization and analysis in this study.

#### BP measurement

Systolic BP will be ascertained at all study visits using the BPTru^® ^device, with six readings performed one minute apart in the arm with the highest reading, and last five readings averaged. These are similar to the methods used in the Canadian health measures survey [56] and the third national health and nutrition examination survey (NHANES III). The BpTRU^® ^automated device has been approved by both the Canadian Hypertension Education Program and the Association for the Advancement of Medical Instrumentation.

#### Outcomes

Because this is an active control trial, we expect improvements in all aspects of care in the control arm--due to both active intervention by stroke specialists within the SPCs and the referring primary care physicians in response to the monthly reminders about each study participant's BP, various secular/temporal influences as they relate to vascular risk reduction, study volunteer and Hawthorne effects, and regression to the mean. The importance of a control group cannot be over-emphasized. For example, in the ESP-CAD trial, we found a 50% absolute improvement in statin management in the control group [35]. Therefore, all continuous measurements will be 'changes' from baseline to six months, comparing the changes achieved in the intervention group with those in the control group.

#### Primary outcome

Because hypertension and dyslipidemia are the most important risk factors for recurrent cardiovascular events in patients with ischemic stroke or TIA [5,57,58], a composite outcome incorporating both has been selected as the primary study outcome. Specifically, the primary outcome is the proportion of subjects who attain 'optimal BP and lipid control'(defined as systolic BP ≤140 mm Hg and fasting LDL cholesterol ≤2.0 mmol/L) at six months according to allocation status.

#### Secondary outcomes

Secondary outcomes will include change in systolic BP at six months versus baseline, proportion achieving BP target (SBP ≤140 mmHg), change in LDL cholesterol at six months versus baseline, proportion attaining LDL cholesterol targets (LDL ≤2.0 mmol/L), proportion attaining total:HDL cholesterol ratios ≤4.0, changes in the cardiovascular disease life expectancy model score [53], changes in other vascular risk factors (waist circumference, body-mass index, self-reported smoking rates, and physical activity), and therapy changes (including number, dosing, and self-reported adherence with antihypertensive and lipid lowering agents). Although differences in clinical events are not anticipated given the short duration of this trial, data will be collected on all-cause hospitalizations, emergency room visits, primary care physician visits, mortality, and clinical events (confirmed by an independent outcome validation committee, blinded to allocation status) such as stroke, TIA, MI, or revascularization. We will also examine humanistic outcomes such as changes in EUROQOL(EQ)-5D [59] and the modified Rankin scale [60] in intervention versus control patients over the course of the trial and will collect direct and indirect costs to conduct a formal cost-effectiveness analysis if the intervention is efficacious. Finally, in order to explore the sustainability of any changes induced by the six-month intervention, we will examine changes in the primary and secondary outcomes at twelve months in both study arms (*i.e*., six months after the intervention stopped). Note that all data will be collected during active follow-up in the twelve-month study period, and further clinical event data will be collected by passive follow-up (for up to five years) through annual linkage to provincial physician billing databases/Canadian Institute for Health Information (CIHI)/provincial vital statistics and registered persons databases after completion of this study as per prior studies by our group [61].

#### Outcomes ascertainment

The primary and secondary outcomes will be collected and analyzed in an independent and blinded manner by research personnel who have not been involved in the patient's care and will be blinded to patient's randomization group and baseline measurements (including BPs). Thus, although patients and their physicians cannot be masked to the fact that they are in the intervention arm, those collecting outcome data and analyzing the data will be blinded.

Systolic BP will be ascertained at baseline and at six months using the BPTru^® ^device. All study-related lab measurements, collected at baseline, six, and 12 months, will be drawn and independently analyzed at one central lab (Dynalife Dx, Edmonton, Alberta, Canada). All survey instruments will be administered locally at baseline, 6, and 12 months, but collated and analyzed at one central lab, the Epidemiology Coordinating and Research (EPICORE) Centre, Division of Cardiology, University of Alberta. Clinical events reported by patients, their family members, or their primary care physicians will be independently validated by the central outcome validation committee that will be blinded to patients' allocated treatment arms. All patients will be asked to sign the appropriate consent and privacy forms needed to permit examination of clinical outcomes, resource use, and vital status at six months and one year (to cross-check and validate data collected at the active follow-up appointments), and at five and ten years, to gain further insights into long-term clinical event rates.

#### Sample size

In a survey of members of the divisions of neurology and general internal medicine at the University of Alberta, we determined that the 'minimal' clinically important difference for this particular intervention to be considered useful was a 10% absolute improvement in the proportion of patients achieving 'optimal BP and lipid control' over and above usual care. After six months, we estimate that no more than 5% of control patients will have attained our composite primary outcome (given that patients who are at goal BP and lipid levels at baseline will be excluded from this study). We calculated our sample size to detect a 10% absolute increase in the primary outcome, set the alpha error rate at 0.05 (2-sided), and the beta error at 0.20 (power 80%); this yielded a minimal necessary sample size of 140 patients per study arm.

The calculated sample size will also provide 80% power (assuming a standard deviation of 15 mm Hg, as recently found in our trial of diabetic hypertensive patients) [34] to detect a 5 mm Hg between-group difference in systolic BP at six months with alpha of 5% for a two-sided test. Based on a review of the literature, consensus of the investigators, and consensus of over two dozen members of the Evidence-Based Recommendations Task Force of the Canadian Hypertension Education Program (surveyed by Dr. McAlister, in his role as Chair of the Central Review Committee for the Evidence-Based Recommendations Task Force of the Canadian Hypertension Education Program]), a 5 mmHg sustained difference in SBP, over and above usual care, would be considered clinically meaningful.

A sample size of 140 patients per group will also provide 85% power (assuming a standard deviation of 0.84 mmol/L, as recently found in our trial in patients with coronary disease) [35] to detect a 0.3 mmol/L between-group difference in fasting LDL cholesterol at six months with alpha of 5% for a two-sided test. This sample size will also provide 85% power (assuming a standard deviation of 14%, as recently found in a trial of 2,687 patients) [53] to detect a 5% between-group difference in projected five-year risk of cardiovascular disease at six months with alpha of 5% for a two-sided test.

One interim analysis is planned, when 140 patients (50% of the projected minimum sample) have had their six-month outcomes ascertained. An independent data and safety monitoring board will examine whether our assumptions about changes in BP and lipid levels (as well as standard deviations) are consistent with the data at that time and will make a recommendation to the study steering committee about whether or not we should continue the study as planned, increase enrollment, or stop the study due to futility. Although we had initially proposed a total sample size of 340 (reflecting a 20% inflation above the minimum necessary sample size to account for potential losses to follow-up), in our most recent trial of 480 patients with coronary artery disease, losses to follow-up were only 3% due to the new province-wide electronic health record in Alberta. Thus, we will examine losses to follow-up in the first 140 patients randomized to determine how much 'inflation factor' we must apply to the sample size to ensure we have outcomes for at least 140 patients per arm at 6 months.

### Statistical analysis

All sociodemographic and clinical characteristics at baseline and follow-up will be summarized using percentages for categorical variables and medians (interquartile range) for continuous variables. Chi square tests will be used to compare the proportion of patients who attain 'optimal BP and lipid control' at six months (because the proportion at baseline will be zero by definition), as well as other binary secondary outcomes such as proportions that achieve target BP and lipid levels. Patient and health system factors associated with meeting BP and lipid goals will be assessed in multivariate analyses. To compare changes in systolic BP, LDL cholesterol, and total:HDL cholesterol ratios between intervention and control, we will use 2-sample independent t-tests. In the event that any baseline characteristics are not balanced between study groups, we will employ multivariate extensions of our primary analytic plan (*i.e*., substituting multiple linear regression for t-test or multiple logistic regression for Chi square test), adjusting for any clinically important (*i.e*., greater than 10% imbalance between arms) or statistically significant (*i.e*., p-value of < 0.10 between arms) differences observed between groups.

All analyses will be by intention to treat. Missing data at the six-month follow-up assessment will be imputed with a 'baseline-observation carried forward' strategy; this approach conservatively assumes that all subjects lost to follow-up have had no change in their BP or lipid levels. As a sensitivity analysis for this assumption, we will also analyze the data in an 'on-treatment' analysis, using only those cases with complete follow-up data for each analysis.

### Data management

All data will be collected using standardized data sheets and data collation, entry, quality assurance, and analysis will be carried out by the EPICORE Centre.

### Ethical considerations

Each patient will be given written information about the study and written informed consent will be obtained prior to study entry. The study protocol was approved by the Health Research Ethics Board, University of Alberta (study ID Pro00001556). The funding for the study is from two peer-reviewed grants. The funding sources (the Alberta Heritage Foundation for Medical Research and the Heart and Stroke Foundation of Canada) had no role in the design of the study and will have no role in the conduct, analysis, interpretation, or reporting of the study, nor access to the data.

## Discussion

We report the protocol for a randomized, controlled trial that aims to determine the effect of a pharmacist case manager to improve control of BP and serum lipids in patients with recent ischemic stroke/TIA. There is a large and universally reported care gap in the prevention of vascular disease--even in SPC attendees--and limited data on interventions proven to improve outcomes in patients with stroke or TIA. Studies in other chronic disorders (*e.g*., coronary disease, heart failure, and diabetes mellitus) offer hope that multifaceted interventions employing pharmacist case managers may improve prescribing/dosing of medications and adherence to medications, resulting in improved risk factor profiles and clinical outcomes. However, it is important to develop and prospectively investigate the role of a pharmacist case management program in concert with current standard of care (*i.e*., SPCs) for survivors of stroke/TIA. Because disease management programs do not always work [36,62,63], and the benefits (if present) are often condition-specific, such programs must be tested in controlled trials, rather than just assuming they are beneficial. Although ultimately the multifaceted program we are proposing will be judged by its impact on clinical events, this study has been designed to test the impact of our program on important processes of care (medication management and adherence) as well as intermediate outcomes (such as BP and cholesterol levels) that are well-validated as predictors of subsequent stroke and cardiovascular events.

## Competing interests

The authors declare that they have no competing interests.

## Authors' contributions

FAM, SRM, and AS conceived the study; FAM and SRM designed the study with input from all authors. FM and MF drafted this manuscript, although all authors provided comments on the drafts and have read and approved the final version.
